# New Insights into Genetic Diversity and Differentiation of 11 Buffalo Populations Using Validated SNPs for Dairy Improvement

**DOI:** 10.3390/genes16040400

**Published:** 2025-03-30

**Authors:** Alfredo Pauciullo, Giustino Gaspa, Carmine Versace, Gianfranco Cosenza, Nadia Piscopo, Meichao Gu, Angelo Coletta, Tanveer Hussain, Alireza Seidavi, Ioana Nicolae, Attawit Kovitvadhi, Qingyou Liu, Jianghua Shang, Jingfang Si, Dongmei Dai, Yi Zhang

**Affiliations:** 1 Department of Agricultural, Forest and Food Science, University of Torino, Grugliasco, 10095 Torino, Italy; giustino.gaspa@unito.it (G.G.); carmine.versace@unito.it (C.V.); 2 Department of Agriculture, University of Napoli Federico II, Portici, 80055 Naples, Italy; giacosen@unina.it; 3 Department of Veterinary Medicine and Animal Production, University of Napoli Federico II, 80137 Naples, Italy; nadia.piscopo@unina.it; 4 National Institute of Biological Sciences, Beijing 102206, China; gumeichao@163.com; 5 Research, Innovation and Selection for Buffalo, 81100 Caserta, Italy; direzione.risbufala@gmail.com; 6 Department of Biological Sciences, Virtual University of Pakistan, Islamabad 44000, Pakistan; tanveer.hussain@vu.edu.pk; 7 Department of Animal Science, Rasht Branch, Islamic Azad University, Rasht 4147654919, Iran; alirezaseidavi@iaurasht.ac.ir; 8 Research and Development Institute for Bovine Breeding, Balotesti, 077015 Bucharest, Romania; ioana_nicolae2002@yahoo.com; 9 Department of Physiology, Kasetsart University, Bangkok 10900, Thailand; fvetawk@ku.ac.th; 10 School of Life Science and Engineering, Foshan University, Foshan 528225, China; qyliu-gene@fosu.edu.cn; 11 Buffalo Research Institute, Chinese Academy of Agricultural Sciences, Nanning 530001, China; jh_shang@163.com; 12 College of Animal Science and Technology, China Agricultural University, Beijing 100193, China; sijingfang@foxmail.com (J.S.); daidm1366@163.com (D.D.); yizhang@cau.edu.cn (Y.Z.)

**Keywords:** river buffalo, swamp buffalo, wild buffalo, genetic diversity, F_ST_, casein

## Abstract

**Background/Objectives:** Buffalo populations exhibit distinct genetic variations influenced by domestication history, geographic distribution, and selection pressures. This study investigates the genetic structure and differentiation of 11 buffalo populations, focusing on five loci related to milk protein (*CSN1S1* and *CSN3*) and fat metabolism (*LPL, DGAT1* and *SCD*). The aim is to assess genetic variation between river, swamp, and wild-type buffaloes and identify key loci contributing to population differentiation. **Methods:** Genetic diversity was analyzed through allele frequency distribution, the Hardy−Weinberg equilibrium testing, and observed (Ho) and expected heterozygosity (He) calculations. Population structure was assessed using principal component analysis (PCA), F_ST_ statistics, and phylogenetic clustering (k-means and UPGMA tree). The silhouette score (SS) and the Davies−Bouldin index (DBI) were applied to determine optimal population clustering. **Results:** Significant genetic differentiation was observed between river and swamp buffaloes (*p* < 0.001). *DGAT1* and *CSN3* emerged as key markers distinguishing buffalo types. The Italian Mediterranean buffalo exhibited the highest genetic diversity (Ho = 0.464; He = 0.454), while the Indonesian, Chinese, and Vietnamese populations showed low heterozygosity, likely due to selection pressures and geographic isolation. The global F_ST_ (0.2143; *p* = 0.001) confirmed moderate differentiation, with closely related populations (e.g., Nepal and Pakistan) exhibiting minimal genetic divergence, while distant populations (e.g., Egypt and Indonesia) showed marked differences, and the Romanian population showed a unique genetic position. **Conclusions:** These findings contribute to a deeper understanding of buffalo genetic diversity and provide a valuable basis for exploiting the potential of this species in the light of future breeding and conservation strategies specific for each buffalo type.

## 1. Introduction

The water buffalo (*Bubalus bubalis*) plays a significant role in global agriculture, particularly in regions such as South Asia, the Mediterranean, and South America. This species, domesticated over 5000 years ago, is essential in providing milk, meat, and draught power in various agro-ecological settings. In particular, buffalo milk, with its high fat content and rich nutrient profile, is a critical resource for dairy industries, contributing significantly to the production of high-quality dairy products like mozzarella cheese, yogurt, butter, and other dairy products [1,2]. The economic importance of buffalo extends beyond its dairy production, as its meat is also highly valued for its tenderness and nutritional qualities, and its adaptability to challenging environments makes it a vital resource in many developing countries [3].

The genetic diversity within the buffalo population is a critical aspect that influences their adaptability, productivity, and overall genetic improvement. This diversity is shaped by centuries of natural selection and human intervention through breeding practices, leading to distinct breeds with unique characteristics. Most of breeds belong to the river type (2n = 50) that has been reared more as a dairy animal than the swamp type (2n = 48), with fewer breeds, which have instead been used more as draught and meat production [1]. Domestication, history, and genetic diversity of both buffalo types have been studied at a genetic level using microsatellites and/or mitochondrial and Y-chromosome DNA markers [4,5,6,7,8,9,10,11] and a genomic level by the 90 K Array SNP chip [12,13] and the whole genome sequencing [14,15]. The application of the most advanced molecular tool helped to support the scenario of two independent domestication events for the river and swamp buffalo [16].

Understanding and harnessing the found genetic variability is essential for improving economically important traits, such as milk yield, meat quality, and disease resistance. Among the key genetic elements influencing these traits are the genes *CSN1S1*, *CSN3*, *DGAT1*, *SCD*, and *LPL*. These genes are known to play vital roles in milk production and quality. The *CSN1S1* (αs1-casein) and *CSN3* (k-casein) genes are involved in the synthesis of casein, the main protein found in milk, which directly affects milk’s nutritional and processing properties [17,18,19,20]. Variations in these genes can significantly influence milk protein content and cheese-making properties, making them important targets for genetic improvement in dairy buffalo [18,21].

The diacylglycerol O-acyltransferase 1 (*DGAT1*) gene is essential in the final step of triglyceride synthesis, a major component of milk fat. Variants in this gene have been associated with variations in milk fat percentage, making it a significant marker for selecting high-fat yielding buffalo breeds [22]. The same gene in cattle is a well-known QTL for fat in meat and milk, demonstrated in many different breeds [23,24,25].

Similarly, the stearoyl-CoA desaturase (*SCD*) gene is another important gene influencing the level of unsaturated fatty acids (FAs) as the SCD enzyme catalyses the insertion of a Δ9 double bound using the saturated FAs as a substrate. Therefore, impacting the FAs composition, SCD is considered a key factor in determining the quality of milk and dairy products [26,27]. In addition, the lipoprotein lipase gene (*LPL*) plays a crucial role in lipid metabolism, influencing the fat content and overall energy balance in buffalo, which are important factors for both milk yield and meat quality [28].

Former studies demonstrated that single nucleotide polymorphisms (SNPs) within these genes have been associated with favorable performances in milk yield and quality, as well as carcass characteristics, thus providing valuable markers for genetic selection and breeding programs [18,19,21,26,29,30], which can improve the overall efficiency and economic returns of buffalo farming.

The aim of the study was to use SNPs in these five genes (*CSN1S1*, *CSN3*, *DGAT1*, *SCD*, and *LPL*) to estimate the genetic relationships, distances, and diversity among 11 buffalo populations, including river (2n = 50), swamp (2n = 48), and wild type, which go from the Mediterranean area to the South East Asia, and to understand how selection differentiated allele frequencies in these key genes for milk and meat traits.

## 2. Materials and Methods

### 2.1. Samples and DNA Isolation

**Figure 1:**
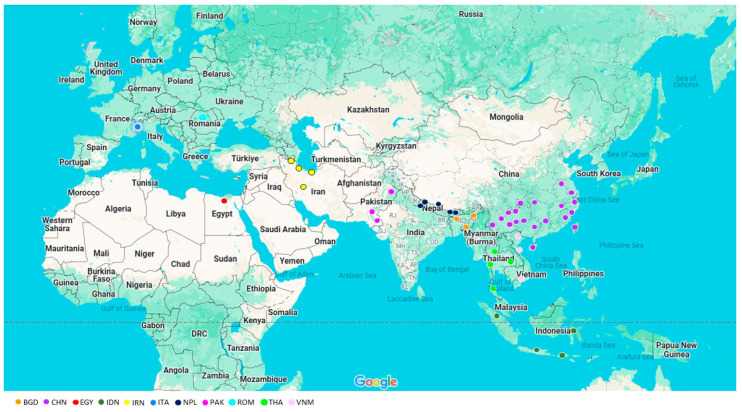
Distribution of buffalo populations under analysis.

A total of 918 domestic buffaloes belonging to 11 populations (48 subpopulations, SPs) were considered in this study. Briefly, populations were indicated by the codes: BDG—Bangladesh (3 SPs, 46 samples); CHN—China (18 SPs, 149 samples); EGY—Egypt (1 SP, 19 samples); IDN—Indonesia (3 SPs, 56 samples); IRN—Iran (4 SPs, 132 samples); ITA—Italy (1 SP, 232 samples); NPL—Nepal (5 SPs, 53 samples); PAK—Pakistan (3 SPs, 27 samples); ROM—Romania (1 SP, 98 samples); THA—Thailand (5 SPs, 91 samples); and VNM—Vietnam (3 SPs, 15 samples). The geographical sampling area is reported in Figure 1, whereas details on subpopulations and their respective animal distributions are listed in Table S1.

Genomic DNA was isolated from hair root samples and from dry blood spots by NucleoSpin^®^ Tissue kit (Macheray-Nagel, Düren, Germany) following the manufacturer’s guidelines. DNA quality and integrity were assessed using a Nanodrop One Spectrophotometer (Thermo-Scientific, Waltham, MA, USA).

### 2.2. PCR Amplification and Genotyping

Standard methods based on PCR (artificially created restriction site (ACRS) and restriction fragment length polymorphism (RFLP)) were used for genotyping the following five SNPs: g.129,635,007A>G at *LPL*; g.32,148,856A>G at the *CSN1S1*; g.31,917,000A>G at the *CSN3*; g.81,685,203G>A at *DGAT1* and g. 21,066,603C>A at *SCD*. SNP positions refer to UOA_WB_1 (GCF_003121395.1) reference genome. Chromosomal location, SNP positions in the gene, genotyping methods, and references are reported in Table 1.

PCR amplifications were carried out using BioRad T100 thermocyclers (BioRad, Hercules, CA, USA) in a total volume of 15 µL containing 50 ng of genomic DNA, 1 × PCR buffer (Promega, Madison, WI, USA), 2.5 mM of MgCl_2_, 5 pmol of each primer, dNTPs, each at 200 µM, and 1 U of Taq DNA Polymerase (Promega). Digestion products were loaded on 2.5% agarose gels for electrophoresis analysis in 0.5 × TBE buffer and stained with SYBR green nucleic acid stain (Lonza Rockland Inc., Rockland, ME, USA).

### 2.3. Statistical Analysis

Within-population variability was estimated by the allele frequencies, gene diversity per locus and population, and observed and expected heterozygosity using the PopGen32 and FSTAT 2.9.3.2 software. The Hardy−Weinberg equilibrium tests were computed for all five loci, and minor allele frequencies were indicated. Using the same software, the Wright’s fixation index (Fis) as a measure of heterozygote deficiency or excess was computed, as well as the diversity between populations was assessed by the pairwise fixation index (F_ST_) and the global F_ST_ (G_ST_). The sequential Bonferroni correction was applied to correct for the effects of multiple tests. Nei’s genetic distances were calculated by 100 permutations. To analyze the population differentiation in more detail, principal component analysis (PCA) was computed grouping both the buffalo types and using the genetic distances matrix combined with clustering analysis by k-means (K = 3 to K = 7) using Phyton software ver 3.13.1. Silhouette and Davies−Bouldin indices were compared for each k-means and used to establish the best clustering separation. Silhouette score measures the ratio between intra-cluster cohesion and inter-cluster separation for each data point, with values ranging from −1 to 1 (where 1 indicates well-defined clusters and −1 indicates poor clustering). In contrast, the Davies−Bouldin index provides a more global measure by averaging similarity between each cluster and its most similar counterpart; DBI values range from 0 to 1, with lower values indicating better clustering. Phyton software was also used to generate the dendogram by UPGMA (Unweighted Pair Group Method with Arithmetic mean), complete linkage, and Ward’s method cluster analysis (1000 bootstraps) [31].

## 3. Results and Discussion

The genetic diversity of 11 buffalo populations was estimated taking into consideration 5 loci of economic interest belonging to milk protein (*CSN1S1* and *CSN3*) and fat metabolism in milk and meat (*LPL*, *DGAT1*, and *SCD*). Polymorphism was detected at all the investigated loci, although the degree of variability differed notably among populations.

**Figure 2:**
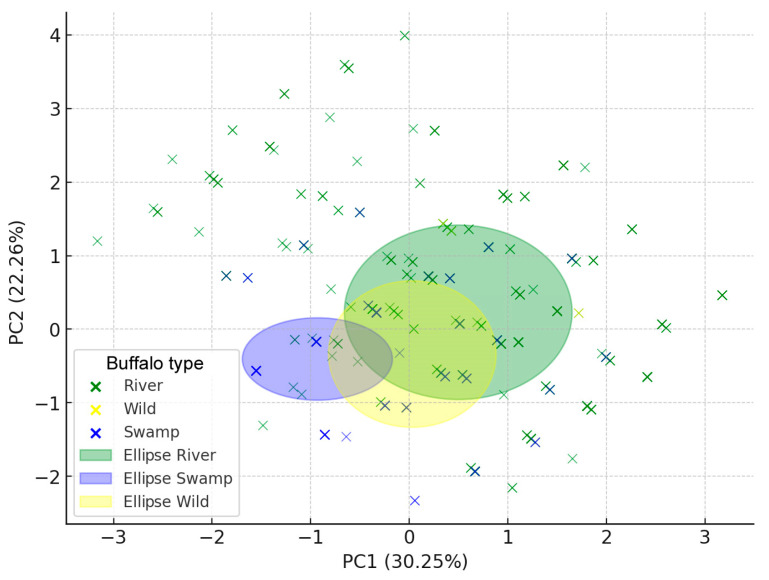
Principal component analysis plot showing the genetic differentiation among buffalo types (river, swamp, and wild). Each point represents an individual, and the ellipses indicate the 95% confidence interval for each group.

Overall, considering all populations in their respective groups (river, swamp, and wild type), the allele frequencies cleraly showed differences among between the river and swamp buffaloes (Figure S1). The χ^2^ test showed extremely low *p*-values (all *p*-values: < 0.001), indicating highly significant differences in allele frequencies among the types and a strong genetic differentiation among the types (Table S2). In particular, the χ^2^ values were very high for the *LPL* g.129,635,007G>A (352.52; *p* = 5.00 × 10^−75^) and *DGAT1* g.81,685,203G>A (324.19; *p* = 6.53 × 10^−69^), thus suggesting that for these genes, the differences between the buffalo populations are likely more marked than for the others. Principal component analysis did not show a very clear clusterization among the subpopulations, although a separation between river (green ellipse) and swamp (blu ellipse) types was evident, whereas the wild type overlapped both river and swamp buffalo populations (Figure 2).

The results were expected. The literature reports that wild buffalo showed ancestry from both river and swamp types, but a much closer relationship to the latter [32]. On the other hands, it was already observed that putative wild buffalo from Kosi Tappu Wildlife Reserve in Nepal [33] clustered strongly with river-type breeds [11]. More recently, a complete genomic landscape study revealed the phylogenetic position of wild Nepalese water buffaloes as a sister taxon of the river type, and similar results were reported also in other studies [34]. Our PCA is consistent with the latter observation since most of our wild types from Nepal (putative *Bubalus arnee*) overlapped with river cluster. Furhermore, the presence of similar allele frequencies in populations like NPL and BGD suggest admixture zones where crossbreeding may have occurred, either naturally in overlapping habitats or through human-mediated practices. Therefore, relatively more recent introgression events from domestic river buffalo in Nepal cannot be excluded, despite the geographical isolation of wild types. This is further supported by the evidence of a spatial hybridization zone documented for some isolated indigenous Nepalese populations (Lime and Parkote) that have shared grazing and breeding areas with possibilities of intercrosses [14].

The PC1 explaind 30% of genetic variance, and the PC2 explained 22% of it. From the PCA loading scores values (Table S2), the *DGAT1* had the highest absolute loading on PC1, meaning among the considered markers it is the most influential gene in explaining the primary genetic variation among the buffalo types, thus confirming the χ^2^ observation. *DGAT1* is well known to be a QTL for fat deposit in carcasses [24] and fat content in milk [23,25], demonstrated in many dairy breeds. This could mean that fat synthesis pathways vary significantly among buffalo populations. Association studies between SNPs at *DGAT1* and milk traits (yield and fat) have been reported in Italian Mediterranean buffalo, Chinese and Anatolian buffalo breeds, and Indian and Brazilian Murrah [35]. This confirms *DGAT1* as a target gene for improvement of milk and meat traits in this species. Similarly, *CSN3* had the highest absolute loading on the PC2, indicating that this gene plays a dominant role in the secondary genetic variation. *CSN3* is a milk protein gene associated with milk quality, coagulation properties, and cheese-making potential [18,20]. In particular, the *CSN3* X2 variant in combination with *CSN1S1* A allele produced higher curd yields primarily due to increased protein content rather than overall milk yield, along with a higher ratio of real to estimated curd yield (RCY/ECY) [19,20]. Therefore, *DGAT1* and *CSN3* can be considered as distinguishing factors playing a role in separating the buffalo types. For instance, casein genes were recently identified as key genes expressed in luminal cells of buffalo mammary gland, which were demonstrated to be the key cell types underlying the divergence between the dairy and non-dairy buffaloes. In this context, caseins exhibit significantly higher expressions in the luminal cells of the dairy than non-dairy buffaloes [15].

Allele frequencies specific for each buffalo population are reported in Table 2. The lowest MAF was 0.113 for the *SCD* g.21,066,603 C allele. A deviation from the HW equilibrium was found in five cases out of 55 calculations (9.09%) (Table 2). This result reflects real population dynamics and can potentially be interpreted due to historical inbreeding (e.g., Indonesia and Romania), small sample sizes (e.g., Vietnam), or selection pressure, as seen at *LPL* (e.g., south-eastern populations like CHN, IDN, THA, and VNM showed inverted MAF).

On average, low genetic diversity (Ho and He) was found in the IDN, VNM, and CHN populations (Table 3). In details, the VNM population resulted monomorphic for four loci (*CSN1S1, CSN3, DGAT1*, and *SCD*) out of five analyzed, and in general, the caseins (*CSN1S1* and *CSN3*) and *SCD* also did not show polymorphism in the Chinese and Indonesian populations; therefore, their He was zero (Table 2 and Table 3).

Conversely, all other populations were polymorphic for all five loci under investigation, and ITA showed the highest Ho (0.464) and He (0.454) (Table 3).

The reduced heterozygosity or even monomorphism could suggest the influence of a selection pressures or past bottleneck events. While the second hypothesis has been excluded by other authors for a gradual and modest decrease in the microsatellite heterozygosity [10], more likely, the natural selection pressure and a strong geographic differentiation with a lack of gene flow can be a possible reason, especially in the swamp type [10]. For instance, the lack of gene flow is likely in the Indonesian buffaloes (IDN) that, coming from the Sulawesi and Sumbawa Island, is an isolated population characterized by a long period of inbreeding. The data confirms the relatively low mutational load recently found by Si et al. [15] in the Indonesian buffalo population.

In contrast, the Italian (ITA) Mediterranean buffalo population showed higher observed (Ho = 0.464) and expected (He = 0.454) heterozygosity, in a general agreement with data of Colli et al. [12] (Ho = 0.381 and He = 0.385) based on SNP array data, but in contrast with data observed nearly 25 years ago (Ho = 0.135 and He = 0.173) using microsatellites [7].

The lower heterozygosity values found by the latter authors was explained with a loss of biodiversity in the Mediterranean buffaloes as a consequence of the decline in the number and increased inbreeding. However, we have to consider that in the last decades, the systematic application of paternity tests and kinship analysis aiming at more performant selection schemes in Italian buffalo also enabled a more efficient control of the consanguinity with a consequent increase of heterozygosity. Moioli et al., [7] and Colli et al., [12] also analyzed Egyptian buffaloes, and our data align more with data based on the SNP array (Ho = 0.395 and He = 0.400) [12].

The global F_ST_ value (G_ST_) was 0.2143 (*p* = 0.001), which means that about 21.5% of the total genetic variability is due to differences between populations and the remaining part is attributed to differences between individuals. This value is consistent with moderate genetic differentiation seen in other livestock studies [36].

Almost all the pairwise F_ST_ values were significant (*p* = 0.001), confirming a considerable level of genetic differentiation among the buffalo populations (Table 4). The lowest values were observed for the comparisons between NPL and BGD populations (0.0025) and between NPL and PAK populations (0.0067), whereas the highest values (>0.60) were found for comparisons involving EGY and IDN (0.6830) or EGY and CHN (0.6351) populations. Pairwise F_ST_ analyses further clarified the genetic landscape. Closely located populations (e.g., those from Nepal, Bangladesh and Pakistan) showed minimal differentiation, while comparisons involving populations such as Egypt and Indonesia revealed markedly higher divergence. These results support the notion that geographic isolation, together with divergent selection pressures, has contributed to the observed genetic structure [11].

In this respect, the Indonesian buffalo population (IDN), coming from the islands of Sulawesi and Sumbawa, has been described as highly inbred and exhibiting higher levels of runs of homozygosity (ROH) compared to other swamp buffalo populations [15].

**Figure 3:**
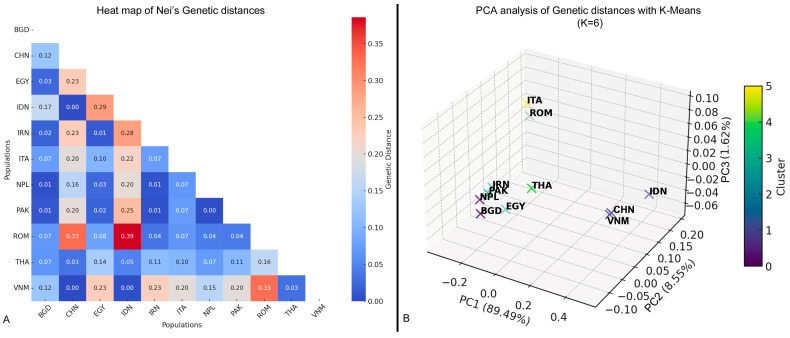
(**A**) Heat map of Nei’s genetic distances. (**B**) 3D representation of the principal component analysis of genetic distances with k-means (K = 6).

Analyses of genetic distances are represented by a heatmap reported in Figure 3A. To have a better representation of the genetic differences and structure among the populations, a principal component analysis was carried out with a clustering analysis for k-means values ranging from 2 to 7, where each cross represents a population cluster evidenced by a different color.

PCA analysis evidenced that the first component (PC1) explained almost 90% of variance among buffalo populations, with PC2 accounting for an additional 8.55%. Some populations (e.g., VNM, CHN, and IDN) clustered together, suggesting a strong genetic similarity. Others, such as ROM, were more distant, indicating a greater genetic divergence. ITA appeared to be positioned close to other Asian populations, which could indicate historical genetic influences (Figure S2A). Moving form a 2D representation to a 3D representation and including also a third component (PC3), a better view of genetic variability was obtained.

Structure analysis within the PCA with K = 2 to K = 5 evidenced that populations, although geographically distant, were often clustered together in more heterogeneous groups (Figure S2B–E). Clastering validity metrics by the comparison of the silhouette score (SS) and the Davis−Bouldin index (DBI) showed that K = 6 gave an optimal balance between internal population cohesion (SS = 0.465) and cluster separability (DBI = 0.268); therefore, populations are better represented (Figure 3B).

Instead, K = 7 showed an over-segmentation of data and a worse performance of the SS and DBI (Table S3 and Figure S2F).

**Figure 4:**
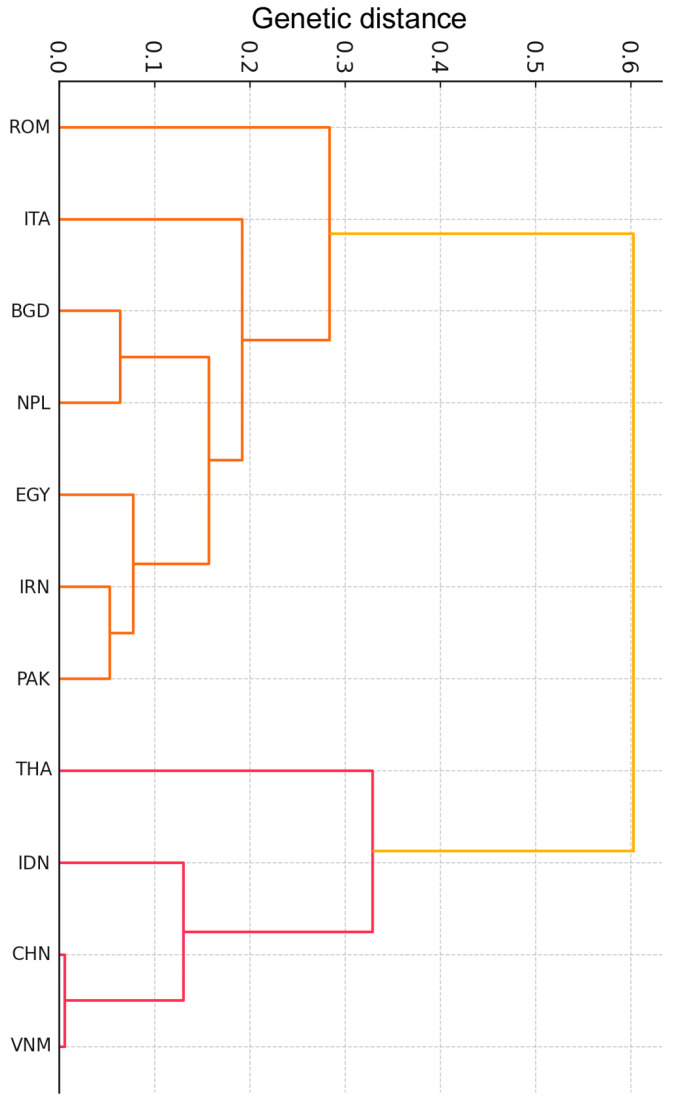
UPGMA consensus tree showing the phylogenetic relationships among the 11 buffalo populations.

The phylogentic analysis was carried out by three different methods (Figure 4 and Figure S3). All dendograms showed consistent results and validated the PCA and k-means (K = 6) analysis, thus confirming the genetic structure and differences among the analysed buffalo populations. In the complex, the genetic diversity observed aligned with the geographic distances separating the different populations from their respective centers of domestication. The high divergence between certain populations (e.g., Egypt vs. Indonesia) and the clustering patterns reinforce the idea of parallel domestication and limited interbreeding between types [12]. A similar observation has also been made using a deeper genomic approach, but with a smaller number of animals, to describe the diversity and the divergence between river and swamp buffaloes [15]. Our data also suggest regional adaptation post-domestication, with selection acting on loci such as *DGAT1* and *CSN3*, which are critical for dairy traits. The consistency of our result confirms that even a few number of SNPs in key genes is useful for structure population studies.

In the UPGMA consensus tree, CHN, VNM, and IDN were always genetically closer and BGD, NPL, and PAK were a joined group, whereas ROM was constantly the most divergent population (Figure 4).

The Romanian buffaloes analyzed in the present study were reared in the Transylvania region (Brașov district) in the area of Șercaia village. They phenotypically exhibited Murrah buffalo traits, especially in the curly horns shape (Figure S4). Although the literature reports that the origin of this buffalo remains uncertain, it is known that, from 1962 to 1990, Murrah buffaloes from India were imported into Bulgaria and spread in Romania to create a new population and upgrade the local buffaloes population after the dramatic decline generated by the Second World War [37].

Currently, the breed is considered as belonging to the Mediterranean type, but it has adapted to colder climates and the regional environment. Therefore, it is indicated as the Mediterranean Carpathian breed [38].

In a recent pilot study on the genetic diversity of German buffaloes, potential relations to herds in other parts of Europe have been investigated, including Romanian buffalo populations (Rom_Serc and Rom_Mera) from Șercaia and Mera villages, respectively [13]. No mixed ancestry was found for Rom_Serc, which was instead separated from the other population located in a former Hungarian village (Mera), now part of Romania. In addition, Rom_Serc has characterized by ancient inbreeding, showing a higher proportion of runs of homozygosity (ROH) compared to the other investigated populations [13]. Therefore, our data confirm the unique genetic position of the ROM population.

Considering the results as a whole, our study confirms that the observed genetic structure of the 11 buffalo populations is the result of a combination of factors including geographic barriers, such as that of island populations (IDN), and environmental adaptation to local agro-ecological conditions (for example, the altitude in Nepal, arid zones in Egypt, or high-humidity in Southeast Asia). Limited gene flow (IDN, VNM, and CHN), structured breeding practices (ITA), and selective pressures for dairy or draft purposes also contributed to differentiate between dairy and non-dairy buffaloes as recently demonstrated by Si et al. [15].

## 4. Conclusions

The use of only five SNPs may appear as a limitation for the resolution of population structure analyses overlooking rare variants or complex genomic regions under selection. However, the use of a gene-targeted strategy in functionally relevant genes offers insights into selection-driven differentiation, especially for applied breeding contexts, and is also cost-effective. This study provides insights into the genetic diversity and differentiation of 11 buffalo populations based on five loci considered as key genes for milk and meat traits. Significant genetic differentiation was observed between river and swamp buffaloes, with *DGAT1* and *CSN3* emerging as key markers of genetic variation. Low genetic diversity was noted in specific populations (IDN, VNM, and CHN), likely due to selection pressures, geographic isolation, and unique genetic position for the Romanian population. These findings contribute to the understanding of buffalo genetic diversity and may be useful for exploiting the potential of this species in the light of future breeding and conservation strategies specific for each buffalo type.

## Figures and Tables

**Table 1:** List of SNPs with relative positions (chromosome, genome, and gene) and genotyping methods applied for the buffalo population’s study.

Chr.	Genome Position	Gene	Location	Reference Allele 5′→3′	Mutation	Genotyping Method	Endonuclease	Reference
3	129,635,007	*LPL*	Exon 1	G	A	RFLP	*DdeI*	Gu et al. [29]
7	32,148,856	*CSN1S1*	Exon 17	A	G	ACRS	*Mbo I*	Pauciullo et al. [19]
7	31,917,000	*CSN3*	Exon 4	A	G	ACRS	*Hinf I*	Pauciullo et al. [19]
15	81,685,203	*DGAT1*	Exon 13	G	A	RFLP	*DdeI*	Gu et al. [30]
23	21,066,603	*SCD*	Promoter	C	A	RFLP	*TaqI*	Gu et al. [26]

**Table 2:** Allele frequencies, chi-square (χ^2^), and *p*-values (P_HW_) of the Hardy−Weinberg (HW) calculations. The deviation from the HW equilibrium is indicated in bold. Population’s sizes are indicated in brackets.

SNP	Allele	Population											
		BGD	CHN	EGY	IDN	IRN	ITA	NPL	PAK	ROM	THA	VNM	All
		(45)	(149)	(19)	(56)	(132)	(232)	(53)	(27)	(98)	(91)	(15)	(918)
*LPL* g.129,635,007G>A	G	0.822	0.225	0.947	0.096	0.943	0.567	0.868	0.944	0.954	0.423	0.233	0.607
	A	0.178	0.775	0.053	0.904	0.057	0.433	0.132	0.056	0.046	0.577	0.767	0.393
	χ^2^	3.080	0.537	0.028	10.061	0.445	2.095	1.933	0.022	0.200	14.404	0.168	152.165
	P_HW_	0.07925	0.46333	0.86577	**0.00151**	0.50448	0.14779	0.16441	0.88149	0.65408	**0.00015**	0.68132	**0.00000**
*CSN1S1* g.32,148,856A>G	A	0.211	0.000	0.474	0.000	0.333	0.306	0.208	0.222	0.194	0.115	0.000	0.196
	G	0.789	1.000	0.526	1.000	0.667	0.694	0.792	0.778	0.806	0.885	1.000	0.804
	χ^2^	1.042	-	0.634	-	0.030	2.768	0.455	0.003	2.426	0.028	-	30.015
	P_HW_	0.30739	-	0.42587	-	0.86154	0.09615	0.49966	0.95699	0.11932	0.86715	-	**0.00000**
*CSN3* g.31,917,000A>G	A	0.178	0.000	0.211	0.000	0.356	0.422	0.283	0.259	0.597	0.324	0.000	0.291
	G	0.822	1.000	0.789	1.000	0.644	0.578	0.717	0.741	0.403	0.676	1.000	0.709
	χ^2^	0.111	-	0.119	-	2.031	0.038	0.009	0.199	13.661	2.738	-	14.925
	P_HW_	0.73939	-	0.72935	-	0.15405	0.84515	0.92261	0.65497	**0.00022**	0.09795	-	**0.00011**
*DGAT1* g.81,685,203G>A	G	0.711	0.990	0.711	0.991	0.708	0.433	0.726	0.667	0.515	0.912	1.000	0.705
	A	0.289	0.010	0.289	0.009	0.292	0.567	0.274	0.333	0.485	0.088	0.000	0.295
	χ^2^	3.961	0.010	0.336	0.004	0.902	0.833	2.196	0.170	0.589	19.978	-	58.428
	P_HW_	0.04656	0.91940	0.56180	1.00000	0.34215	0.36148	0.13834	0.67974	0.44283	**0.00004**	-	**0.00000**
*SCD* g.21,066,603C>A	C	0.044	0.000	0.079	0.000	0.140	0.246	0.123	0.167	0.107	0.033	0.000	0.113
	A	0.956	1.000	0.921	1.000	0.860	0.754	0.877	0.833	0.893	0.967	1.000	0.887
	χ^2^	0.070	-	0.091	-	1.245	1.067	0.948	0.825	1.338	0.088	-	158.077
	P_HW_	0.79056	-	0.76322	-	0.26443	0.30162	0.33020	0.36353	0.24739	0.76717	-	**0.00000**

**Table 3:** Average heterozygosity calculated for the five loci and gene diversity parameters per locus and population.

	**Average heterozygosity**										
	**BGD**	**CHN**	**EGY**	**IDN**	**IRN**	**ITA**	**NPL**	**PAK**	**ROM**	**THA**	**VNM**
Observed	0.244	0.070	0.274	0.021	0.320	0.464	0.294	0.341	0.355	0.235	0.067
Expected	0.286	0.074	0.306	0.046	0.334	0.454	0.318	0.317	0.316	0.272	0.074
	**Expected heterozygosity per *locus* and population**										
	**BGD**	**CHN**	**EGY**	**IDN**	**IRN**	**ITA**	**NPL**	**PAK**	**ROM**	**THA**	**VNM**
*LPL* g.129,635,007G>A	0.296	0.350	0.102	0.177	0.108	0.492	0.232	0.107	0.088	0.492	0.371
*CSN1S1* g.32,148,856A>G	0.337	0.000	0.515	0.000	0.446	0.426	0.332	0.352	0.314	0.205	0.000
*CSN3* g.31,917,000A>G	0.295	0.000	0.342	0.000	0.461	0.489	0.410	0.390	0.483	0.440	0.000
*DGAT1* g.81,685,203G>A	0.417	0.020	0.424	0.052	0.415	0.492	0.402	0.453	0.502	0.162	0.000
*SCD* g.21,066,603C>A	0.086	0.000	0.149	0.000	0.242	0.371	0.217	0.282	0.192	0.064	0.000
	**Wright’s fixation index (Fis) as a measure of heterozygote deficiency or excess**										
	**BGD**	**CHN**	**EGY**	**IDN**	**IRN**	**ITA**	**NPL**	**PAK**	**ROM**	**THA**	**VNM**
*LPL* g.129,635,007G>A	0.254	0.060	−0.029	0.403	−0.056	−0.095	0.186	−0.040	−0.043	0.397	0.103
*CSN1S1* g.32,148,856A>G	0.148	-	0.182	-	0.015	0.109	0.092	−0.053	0.156	−0.017	-
*CSN3* g.31,917,000A>G	−0.048	-	0.077	-	0.124	0.013	−0.013	−0.139	−0.374	−0.173	-
*DGAT1* g.81,685,203G>A	0.291	−0.007	0.131	0.000	0.083	−0.060	0.202	0.019	−0.078	0.456	-
*SCD* g.21,066,603C>A	−0.034	-	−0.059	-	−0.096	−0.068	−0.130	−0.182	−0.115	−0.029	-

**Table 4:** Pairwise F_ST_ values (below the diagonal) and related *p*-values (above the diagonal) among the 11 domestic buffalo populations.

	BGD	CHN	EGY	IDN	IRN	ITA	NPL	PAK	ROM	THA	VNM
**BGD**		***	NS	***	***	***	NS	NS	***	***	***
**CHN**	0.4562		***	NS	***	***	***	***	***	***	NS
**EGY**	0.0382	0.6351		***	NS	***	NS	NS	***	***	***
**IDN**	0.4912	0.0562	0.6830		***	***	***	***	***	***	NS
**IRN**	0.0350	0.4640	0.0104	0.4669		***	NS	NS	***	***	***
**ITA**	0.0992	0.3308	0.1163	0.3183	0.0983		***	***	***	***	***
**NPL**	0.0025	0.4722	0.0331	0.4879	0.0095	0.0886		NS	***	***	***
**PAK**	0.0095	0.5786	0.0251	0.6122	0.0024	0.0890	0.0067		*	***	***
**ROM**	0.1259	0.5771	0.1345	0.5654	0.0619	0.0930	0.0813	0.0732		***	***
**THA**	0.1410	0.1777	0.2376	0.2059	0.1918	0.1420	0.1415	0.1971	0.2598		**
**VNM**	0.2941	0.0194	0.4495	0.0785	0.3462	0.2521	0.3057	0.3882	0.4440	0.0999	

NS—non significant; * *p* < 0.001; ** *p* < 0.0001; *** *p* < 0.00001.

## Data Availability

The data presented in this study are available on request from the corresponding author.

## Supplementary Materials

The following supporting information can be downloaded at: https://www.mdpi.com/article/10.3390/genes16040400/s1, Figure S1: Allele frequencies at the loci for the buffalo populations grouped per type; Figure S2: 2D and 3D representations of the principal component analysis of genetic distances among the 11 buffalo populations with different k-means (from K = 2 to K = 7); Figure S3: Consensus trees showing the phylogenetic relationship among the 11 buffalo populations performed by complete linkage and Ward’s methods; Figure S4: Example of Romanian buffalo. Samples collected at Șercaia—Research and Development Station for Buffalo Breeding (Brașov district, Transylvania region); Table S1: Populations and subpopulations of analyzed buffaloes; Table S2: Chi-square and relative *p*-values and loading scores of the PCA; Table S3. Clustering validity metrics from K = 2 to K = 7 by the silhouette score (SS) and the Davis−Bouldin index (DBI).

## Abbreviations

The following abbreviations are used in this manuscript:

DBI	Davies−Bouldin index
MAF	minor allele frequency
PCA	principal component analysis
PCR	polymerase chain reaction
QTL	quantitative trait locus
RFLP	restriction fragment length polymorphism
ROH	runs of homozygosity
SNP	single nucleotide polymorphism
SS	silhouette score
UPGMA	unweighted pair group method with the arithmetic mean
