# Supplementary material for: New Insights into Genetic Diversity and Differentiation of 11 Buffalo Populations Using Validated SNPs for Dairy Improvement
Source: Genes (Basel). 2025 Mar 30;16(4):400. doi: 10.3390/genes16040400 (PMC12026637; doi:10.3390/genes16040400)
Supplement: Supplementary file 1 [file genes-16-00400-s001.zip › Figure S2.pdf]

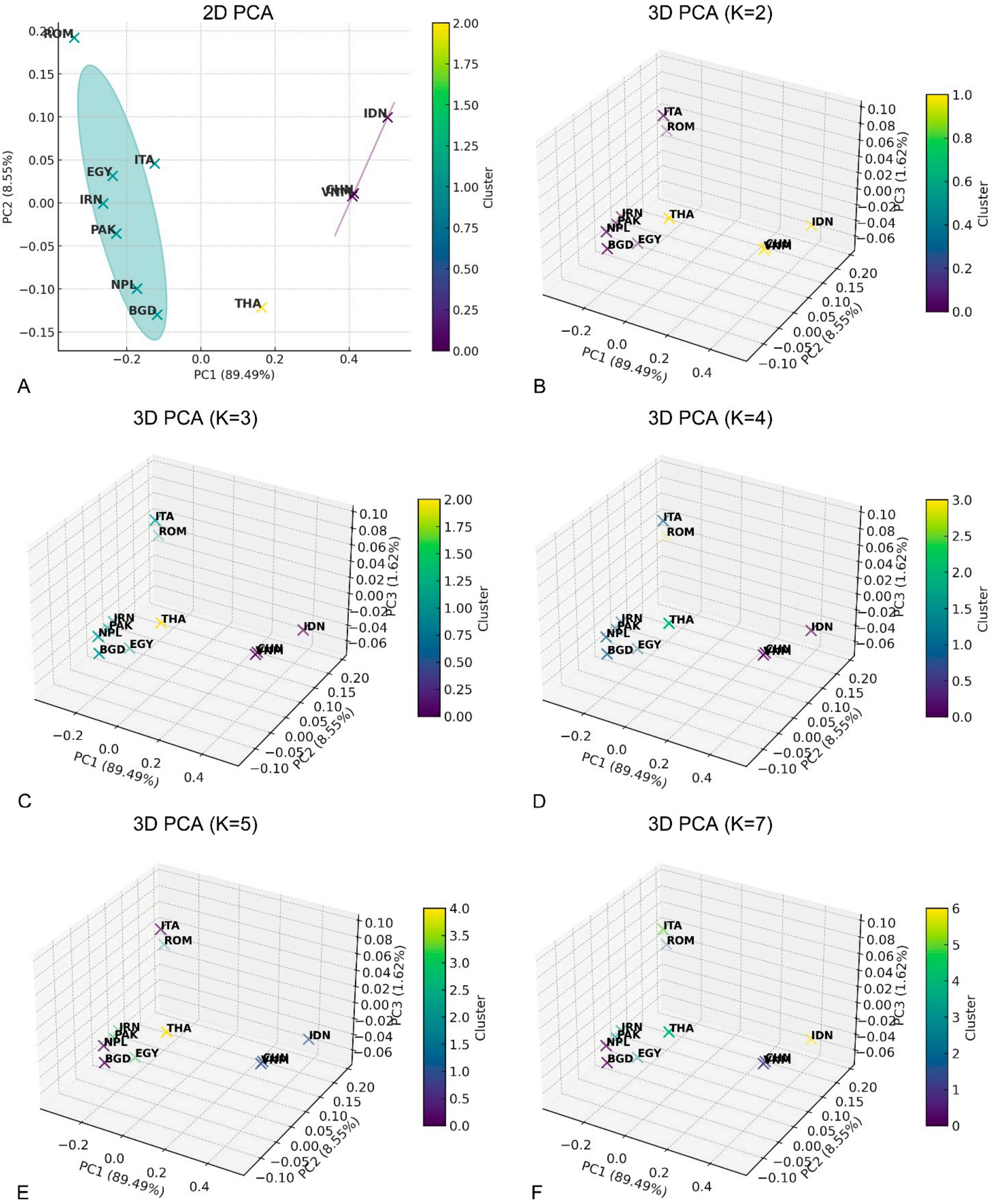

Figure S2. 2D and 3D representation of the Principal Component Analysis of genetic distances among the 11 buffalo populations with different K-means (from K=2 to K=7).
