# Supplementary material for: New Insights into Genetic Diversity and Differentiation of 11 Buffalo Populations Using Validated SNPs for Dairy Improvement
Source: Genes (Basel). 2025 Mar 30;16(4):400. doi: 10.3390/genes16040400 (PMC12026637; doi:10.3390/genes16040400)
Supplement: Supplementary file 1 [file genes-16-00400-s001.zip › Figure S3.pdf]

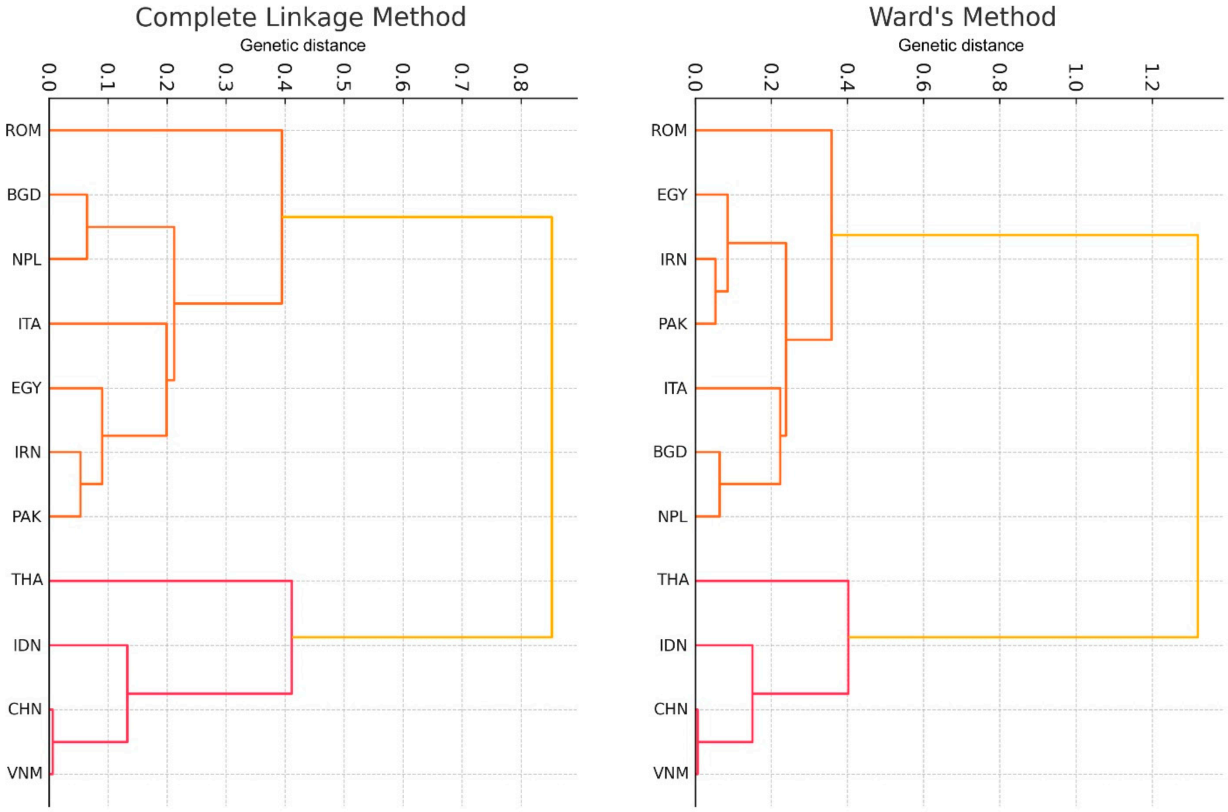

Figure S3. Consensus trees showing the phylogenetic relationship among the 11 buffalo populations done by complete linkage and Ward's methods.
