# Supplementary material for: New Insights into Genetic Diversity and Differentiation of 11 Buffalo Populations Using Validated SNPs for Dairy Improvement
Source: Genes (Basel). 2025 Mar 30;16(4):400. doi: 10.3390/genes16040400 (PMC12026637; doi:10.3390/genes16040400)
Supplement: Supplementary file 1 [file genes-16-00400-s001.zip › Figure S4.pdf]

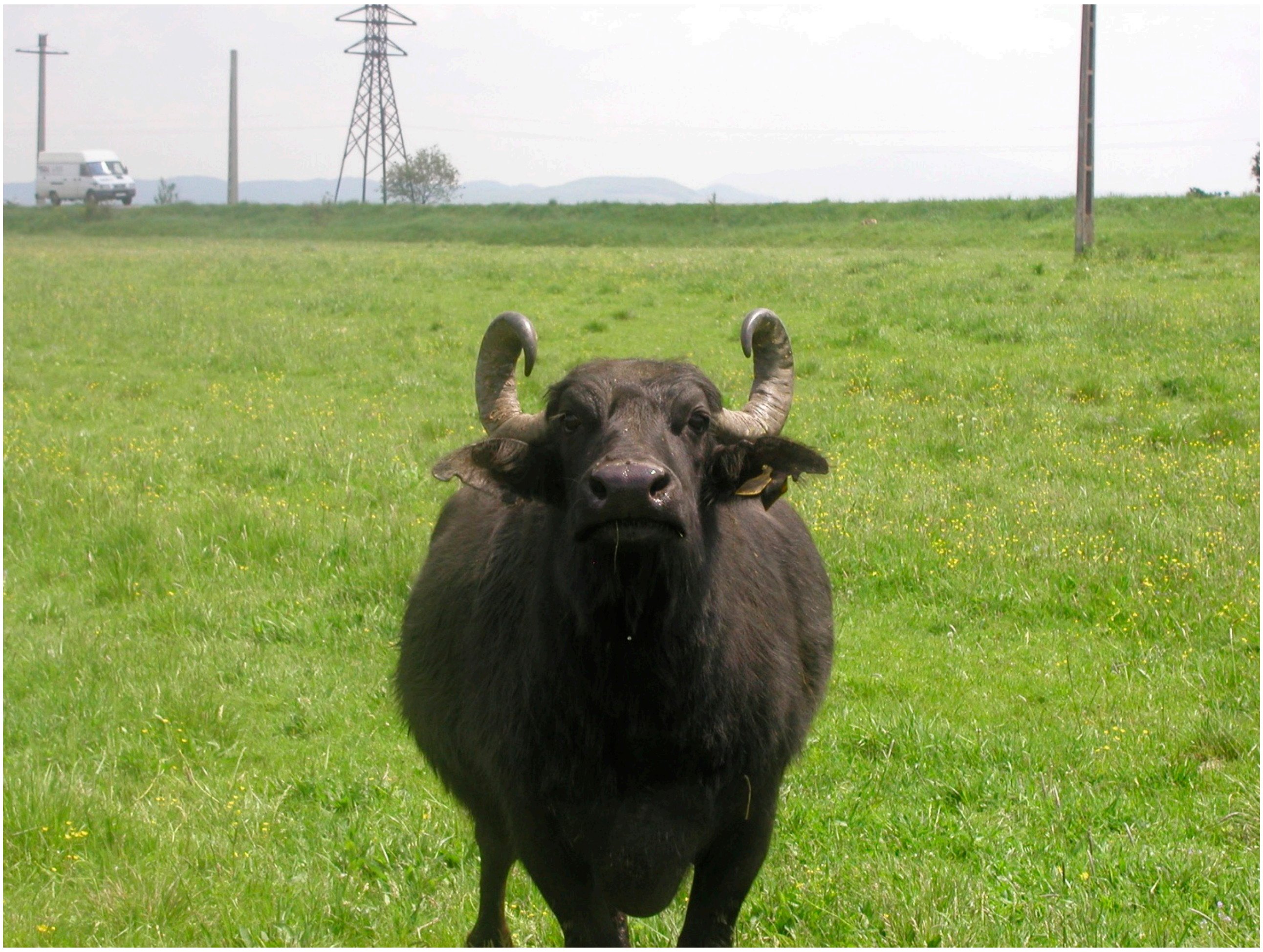

Figure S4. Example of Romanian buffalo. Samples collected at Șercaia - Research and Development Station for Buffalo Breeding (Brașov district, Transylvania region).
