# Supplementary material for: New Insights into Genetic Diversity and Differentiation of 11 Buffalo Populations Using Validated SNPs for Dairy Improvement
Source: Genes (Basel). 2025 Mar 30;16(4):400. doi: 10.3390/genes16040400 (PMC12026637; doi:10.3390/genes16040400)
Supplement: Supplementary file 1 [file genes-16-00400-s001.zip › Table S2.pdf]

Table S2. Chi-square and relative p-values. Loading scores of the PCA.

**Locus**	**Chi-square**	**p-value**	**Loading score**	
			**PC1**	**PC2**
LPL g.129,635,007G>A	352.52	5.00 E-75	-0.45793	-0.30444
CSN1S1 g.32,148,856A>G	147.77	6.12 E-31	-0.47742	0.498626
CSN3 g.31,917,000A>G	181.83	3.01 E-38	-0.43823	0.566678
DGAT1 g.81,685,203G>A	324.19	6.53 E-69	0.508814	0.415527
SCD g.21,066,603C>A	109.63	8.73 E-23	-0.33381	-0.40608

Degree of freedom: 4
