# Supplementary material for: New Insights into Genetic Diversity and Differentiation of 11 Buffalo Populations Using Validated SNPs for Dairy Improvement
Source: Genes (Basel). 2025 Mar 30;16(4):400. doi: 10.3390/genes16040400 (PMC12026637; doi:10.3390/genes16040400)
Supplement: Supplementary file 1 [file genes-16-00400-s001.zip › Table S3.pdf]

Table S3. Clastering validity metrics from K=2 to K=7 by Silhouette score (SS) and Davis-Bouldin index (DBI).

K	SS	DBI	Analysis
2	0.665	0.412	Very broad division into two generic groups, useful for macro comparisons.
3	0.463	0.289	Identified three main groups, but some clusters are still very large.
4	0.465	0.276	Showed clearer separation of populations although some clusters are still large.
5	0.457	0.270	More detail in the separation, but some clusters may be too fragmented.
6	0.465	0.268	Excellent balance between separability and internal cohesion.
7	0.436	0.209	Over-segmentation of data and clusters become too small and less meaningful.
