# Supplementary figures and images for: New Insights into Genetic Diversity and Differentiation of 11 Buffalo Populations Using Validated SNPs for Dairy Improvement
Source: Genes (Basel). 2025 Mar 30;16(4):400. doi: 10.3390/genes16040400 (PMC12026637; doi:10.3390/genes16040400)

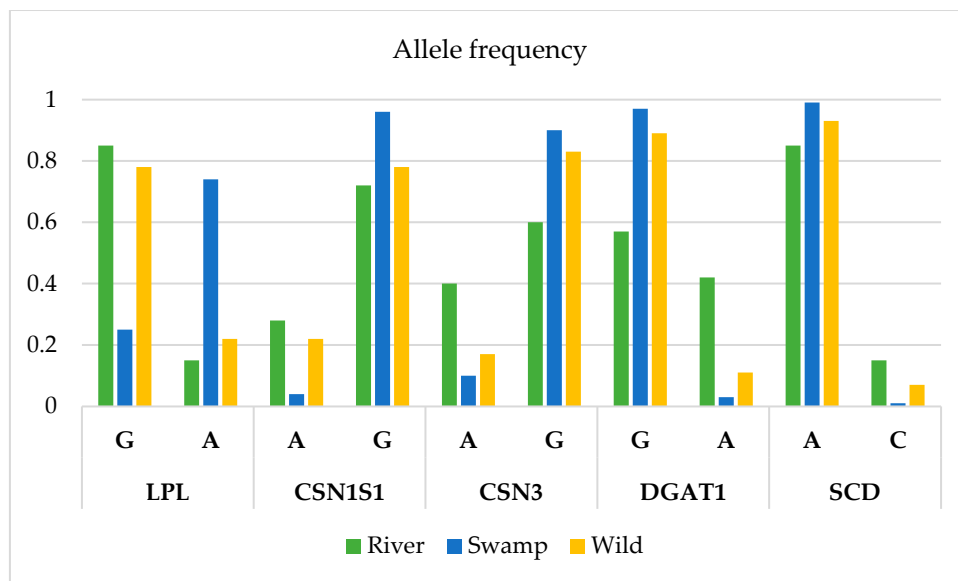

**Figure S1.** Allele frequencies at the loci for the buffalo populations grouped per type.

Supplement: Supplementary file 1 [file genes-16-00400-s001.zip › Figure S1.pdf]
